# Nerve-Glial antigen 2: unmasking the enigmatic cellular identity in the central nervous system

**DOI:** 10.3389/fimmu.2024.1393842

**Published:** 2024-07-29

**Authors:** Marta Bottero, Giada Pessina, Caterina Bason, Tiziana Vigo, Antonio Uccelli, Giovanni Ferrara

**Affiliations:** ^1^ IRCCS Ospedale Policlinico San Martino, Genoa, Italy; ^2^ Department of Experimental Medicine, University of Genoa, Genoa, Italy; ^3^ Department of Neurology, Rehabilitation, Ophthalmology, Genetics, Maternal and Child Health, University of Genoa, Genoa, Italy

**Keywords:** NG2, oligodendrocyte progenitor cells, pericytes, immune cells, neuroinflammation, neurodegeneration

## Abstract

Chondroitin sulfate proteoglycans (CSPGs) are fundamental components of the extracellular matrix in the central nervous system (CNS). Among these, the Nerve-Glial antigen 2 (NG2) stands out as a transmembrane CSPG exclusively expressed in a different population of cells collectively termed NG2-expressing cells. These enigmatic cells, found throughout the developing and adult CNS, have been indicated with various names, including NG2 progenitor cells, polydendrocytes, synantocytes, NG2 cells, and NG2-Glia, but are more commonly referred to as oligodendrocyte progenitor cells. Characterized by high proliferation rates and unique morphology, NG2-expressing cells stand apart from neurons, astrocytes, and oligodendrocytes. Intriguingly, some NG2-expressing cells form functional glutamatergic synapses with neurons, challenging the long-held belief that only neurons possess the intricate machinery required for neurotransmission. In the CNS, the complexity surrounding NG2-expressing cells extends to their classification. Additionally, NG2 expression has been documented in pericytes and immune cells, suggesting a role in regulating brain innate immunity and neuro-immune crosstalk in homeostasis. Ongoing debates revolve around their heterogeneity, potential as progenitors for various cell types, responses to neuroinflammation, and the role of NG2. Therefore, this review aims to shed light on the enigma of NG2-expressing cells by delving into their structure, functions, and signaling pathways. We will critically evaluate the literature on NG2 expression across the CNS, and address the contentious issues surrounding their classification and roles in neuroinflammation and neurodegeneration. By unraveling the intricacies of NG2-expressing cells, we hope to pave the way for a more comprehensive understanding of their contributions to CNS health and during neurological disorders.

## Introduction

Nerve-glial antigen 2 (NG2, UniProtKB - Q6UVK1), also known as chondroitin sulfate proteoglycan 4 (CSPG4), high molecular weight melanoma-associated antigen (HMW-MAA), or melanoma chondroitin sulfate proteoglycan (MCSP), has been the subject of extensive research over the past four decades. It is encoded by the CSPG4 gene, which produces a 250–270 kDa surface protein spanning the cell membrane: the extracellular portion contains chondroitin sulfate glycosaminoglycan chains, disulfide bonds, proteolytic cleavage sites, and hormone binding sites; while the intracellular domain acts as a receptor for extracellular signals (1–3). NG2 has been implicated in various cellular functions, playing a significant role in cell proliferation, migration, and differentiation. The expression of NG2 is regulated by several signaling pathways, such as Notch, Hedgehog, and Wnt pathways. Under normal conditions, NG2 is expressed by multiple cell types, including oligodendrocyte progenitor cells (OPCs), pericytes, and immune cells; in addition, NG2 expression is also found in neurons, mesenchymal stem cells, endothelial cells, osteoblasts, melanocytes, and smooth muscle cells (4–13). However, in pathological conditions, elevated NG2 expression levels are also observed in glioblastoma and tumor cells associated with metastatic formation in soft-tissue sarcoma and melanoma (14, 15). Although the scientific community does not express an explicit consensus on the identity of NG2-expressing cells, there is consistent evidence supporting the notion that, in the CNS, only a small portion of parenchymal cells express NG2 (referred to as OPCs), as well as vascular cells (named activated pericytes). In response to neuroinflammation and neurodegeneration, OPCs actively proliferate, migrate from CNS neurogenic areas (germinal zones) (16–21), and remyelinate damaged areas by differentiating into mature oligodendrocytes (22–26). Moreover, the CNS is known to respond rapidly to demyelinating insults by regenerating oligodendrocytes for remyelination around the glial scar; this regenerative process is mediated by OPCs (6, 25, 27). However, in the glial scar, a large number of NG2-expressing cells do not differentiate into mature myelin-producing oligodendrocytes, resulting in misinterpretation of their real function (28, 29) (for a detailed discussion, refer to the “OPCs” section). On the other hand, pericytes are multi-functional mural cells that display stem cell-like properties and are localized at the abluminal side of the CNS perivascular space, at the level of microvessels. They play several crucial roles: development and maintenance of the neurovascular unit (NVU), regulation of the neurovascular system (blood vessel formation, cerebral blood flow, vascular stability), trafficking of inflammatory cells, and clearance of toxic waste products from the CNS parenchyma (12, 30–32) (for a detailed discussion, refer to the “Pericytes” section). Interestingly, recent studies have shown that NG2 is expressed in CNS-infiltrating macrophages, suggesting a potential involvement of the NG2 proteoglycan in modulating their functions, such as phagocytosis, neuroinflammation, and tissue repair. Thisrepresents a significant advancement in the understanding of the NG2 role within the CNS because macrophages, as part of the immune system, play a crucial role in maintaining CNS health and in responding to various pathological conditions, such as neuroinflammatory diseases and neurological disorders (for a detailed discussion, refer to the “Immune cells” section). Consistently, the seminal work of Nishiyama et al.,in 2001 has demonstrated that NG2 expression is transiently upregulated on activated myeloid immune cells (macrophages/microglia) during the chronic stage of an excitotoxic lesion in the adult CNS (33). Since then, numerous studies from several research groups have described a prominent expression of NG2 by immune cells (34–39). Other relevant papers have demonstrated that NG2-expressing cells show some electrophysiological properties in distinct CNS areas and at different developmental stages, indicating that those cells may play a role in the regulation of the neuronal network (40). Altogether, these observations draw an intricate picture of the identity of NG2-expressing cells, creating a confounding storm of information. Of note that the very name “nerve-glial” of NG2 indicates an unclear identification of the cell type on which it is expressed, merging neurons and glial cells, perhaps highlighting the technical difficulties in cellular identification. Therefore, in this review article, we will try to harmonize the scientific information about NG2, and we will address new insights on the NG2 structure, expression, role during neuroinflammation/neurodegeneration, and the methods of investigation used to describe the roles of NG2-expressing cells.

## The structure and functional domains of NG2

### The structure of NG2

**Figure 1:**
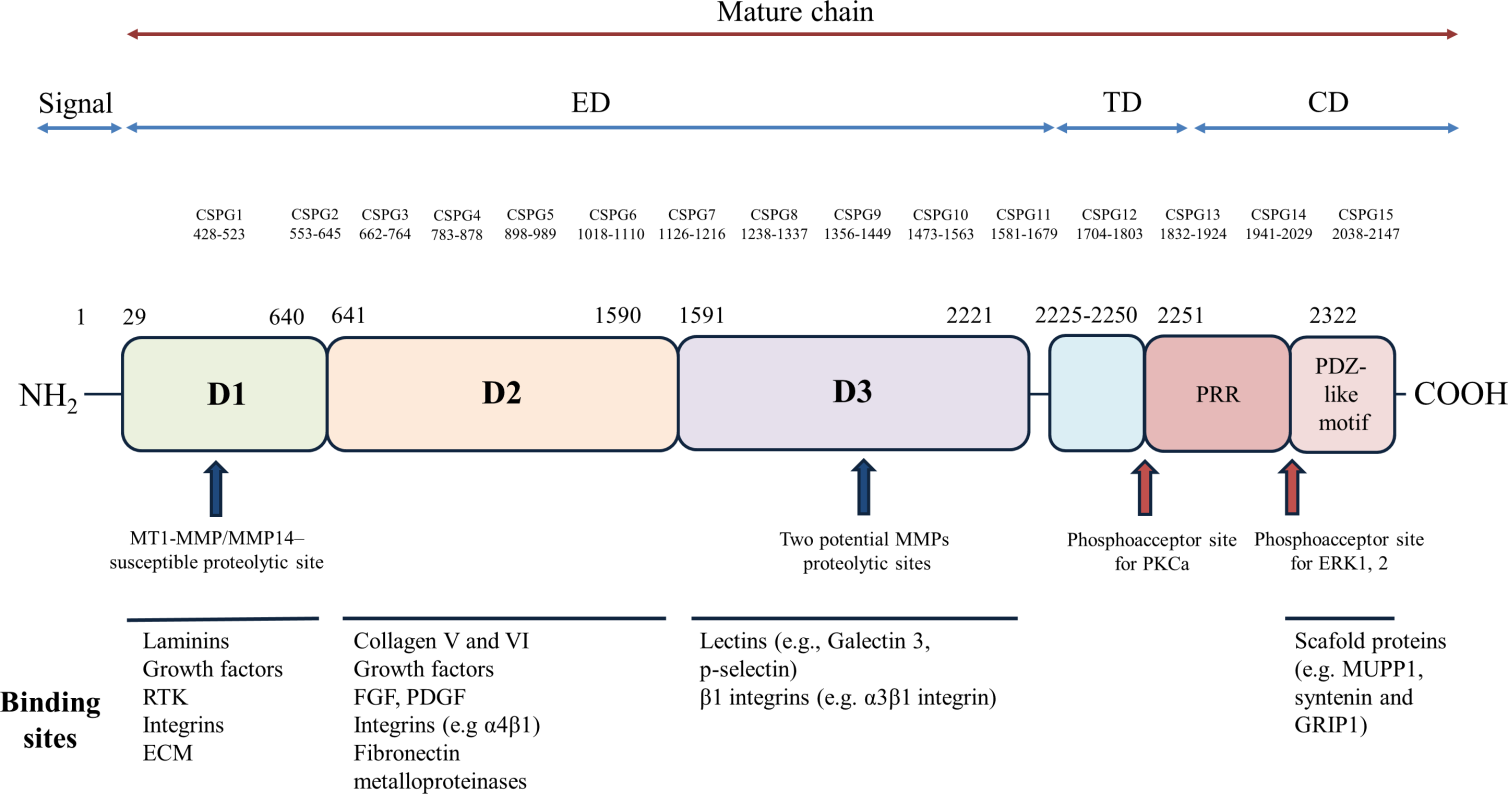
Structural organization and functional domains of NG2. The NG2 protein comprises a large extracellular region (amino acids [aa] 1–2221), a transmembrane domain, and a cytoplasmic tail. The extracellular region is further subdivided into three domains: D1, D2, and D3. The D1 domain, stabilized by disulfide bonds, harbors a single chondroitin sulfate (CS) chain and two laminin G-type globular domains (L1 and L2), which may serve as Ca++-mediated receptors for ECM molecules, including laminins. The D2 domain features a tandem array of approximately 15 CS attachment motifs, multiple CS-GAG binding sites, and a single CS-GAG chain known to interact with integrins and matrix metalloproteinases (MMPs). The D3 domain contains various proteolytic cleavage sites susceptible to MMPs or other proteases. Additionally, cadherin-like sequences are dispersed throughout the extracellular region. A 25-amino acid transmembrane region connects the extracellular and cytoplasmic domains. The cytoplasmic tail encompasses a proline-rich region (PRR), potentially involved in protein-protein interactions, and a PDZ binding domain, which plays a role in protein scaffolding by binding proteins such as synthenin, MUPP1, and GRIP1. Two distinct phosphorylation sites are present in NG2: a PKCa phosphoacceptor site at threonine residue 2256 (Thr2256) and an ERK1/2 phosphoacceptor site at threonine residue 2314 (Thr2314). The extracellular domain of NG2 harbors binding sites for a diverse array of molecules, including ECM proteins, growth factors, integrins, lectins, and matrix metalloproteinases. The major molecules interacting with each domain of NG2 are depicted schematically. CS, chondroitin sulfate; ED, extracellular domain; TM, transmembrane domain; CD, cytoplasmic domain; D1, D2 and D3, domains of the extracellular portion; L1 and L2, 2 contiguous Laminin-G–like domains; PRR, proline-rich region; PDZ, PDZ binding domain; CSPG, repetitive regions. RTK, receptor tyrosine kinase; ECM, extracellular matrix; FGF, fibroblast growth factors; PDGF, platelet-derived growth factor; PKC, protein kinase C; ERK, extracellular signal-regulated kinases; MUPP1, multi-PDZ-domain protein1; GRIP1, Glutamate Receptor Interacting Protein 1.

Proteoglycans (PGs) are a group of molecules that contain glycosaminoglycan chains (GAGs) covalently linked to their core proteins. A large number of these molecules are found in the extracellular matrix or on the cell surface, and they have been implicated in different cell functions, including cell-matrix and cell-cell interactions, as well as interactions with extracellular ligands (41, 42). GAGs, like other polysaccharides, are generated through the polymerizing action of different enzymes which link repeated disaccharides creating individual complex structures. In contrast to glycoproteins that also exhibit carbohydrate side chains, the covalently attached GAG side chains make up most of the molecular weight of PGs and are principally responsible for most of their physiological functions (43). In this context, NG2 is a PG translationally modified by the addition of chondroitin sulfate GAG chains of alternating sugars (N-acetylgalactosamine and glucuronic acid) and disulfide bonds, and its gene encodes a surface type I transmembrane core protein of about 250–270 kDa (44, 45). NG2 contains numerous glycosylation sites and up to 3 putative GAG attachment sites, but only one of these sites, at serine 999 (in humans at position 995), is engaged for the linking (46). Characterizations on the composition of the attached oligosaccharides and GAGs have identified the 2-acetamido-2-deoxy-3-O-(b-D-gluco-4-enepyranosyluronic acid)-b-4-O-sulfo-D-galactose and 2-acetamido-2-deoxy-3-O-(b-D-gluco-4-enepyranosyluronic acid)-b-6-O-sulfo-D-galactose as the prevalent oligosaccharides, while the predominant GAGs are chondroitin sulfate A and B isomers of about 30–60 kDa (47, 48). As indicated in **Figure 1**, molecular and protein-ligand binding experiments have demonstrated that NG2 consists of a flexible rod-like domain separated by two globular regions located at the N-terminal (D1 domain) and C-terminal (D3 domain) ends of the NG2 ectodomain (49). Subsequent characterizations have revealed that the two globular regions are linked by an intramolecular disulfide bond, composed of a flexible segment of alternating α-helical and β-sheet regions (D2 domain) (50). In addition, the D2 domain is considered the putative linking area of two main ligands of NG2, namely collagens V and VI (49). The conformational structure of NG2 is integral to its functions, and NG2 displays an extracellular domain, a single transmembrane region, and an intracellular domain. The extracellular domain is adorned with chondroitin sulfate chains and interacts with a variety of extracellular matrix proteins, growth factors, and signaling molecules. This interactions are critical for the regulation of cell adhesion, migration, and proliferation, which are vital processes in neural development and tissue repair (51). Particularly, the extracellular domain interacts with the extracellular environment, while the intracellular domain engages in intricate intracellular signaling pathways, playing a pivotal role in transducing signals from the extracellular environment into the cell. The intracellular domain contains binding sites for numerous proteins and kinases, including focal adhesion kinase (FAK) and protein kinase C (PKC), which are associated with cell adhesion, migration, and survival (1, 45, 49, 52). The exact mechanisms by which the NG2 intracellular domain modulates these signaling pathways are still under investigation, but it is clear that this domain serves as a key mediator of cell responses to external cues. By transducing signals related to growth and survival, NG2 participates in processes like myelination, tissue repair, and immune responses within the CNS.

### The functional domains of NG2

NG2 is found in progenitors and terminally differentiated cells and its role in cell communication is supported by its characterization as a functional receptor involved in cell-cell interactions. Moreover, its ability to respond to different harmful stimulations might be important in neurodegeneration and neuroinflammation (for a detailed discussion, refer to the “The role of NG2 expressing cells in neurological disorders” section). In NG2-dependent signal transduction, the short intracellular domain mediates the activation of intracellular signaling pathways related to FAK and MAP kinase pathways. These pathways regulate important cellular functions, such as cell proliferation, migration, invasion, cytoskeletal reorganization, and survival (8). In addition, NG2 plays a role as a co-receptor for PDGFRα to activate FAK and MAP kinase pathways (53, 54). The NG2 cytoplasmic domain contains different binding sites for multi-PZD domain protein 1, facilitating the physical interaction of NG2 with the key structural and/or signaling components in the CNS (7), and for the synaptic protein GRIP1 and syntenin-1, important for the NG2-mediated cellular migration (55, 56). In addition, as shown in **Figure 1**, it is shown the presence of two different sites that are phosphorylated by PKCα and ERK in Thr 2256 and 2314 residues, respectively (57). The cell surface localization of the protein is followed by β1 integrin binding to the NG2 extracellular domain, which also contains binding sites for collagens II, V, and VI, galectin, laminin, and tenascin (58). Moreover, recent studies have shown that the sequential cleavage of NG2 mediated by α and γ secretase (so-called NG2 shedding) results in the release of the extracellular and intracellular domains, which act as biologically functional molecules regulating neuronal networks (23). Several studies have suggested a role for NG2 in promoting the proliferation and motility characteristics of both normal progenitor cells and malignant tumor cells (59, 60). As a membrane-spanning molecule, NG2 affects cell proliferation and migration via interactions with both extracellular and intracellular binding partners. The extracellular portion of NG2 plays a role in the interaction with growth factors, in the binding to growth factor receptors (53, 54), in the interactions with extracellular matrix ligands (as collagen VI) (58, 61, 62), and in driving signaling complexes with α3β1-integrin and galectin-3 (52, 63, 64). The cytoplasmic domain of NG2 is involved in the activation of GTPases (Rho, Rac and Cdc42) (65), as well as in the anchorage to the membrane via the PDZ-containing scaffolding protein GRIP1 (56, 66). The NG2 function may be regulated by its post-translational modification, playing an important role in the interaction of NG2 with extracellular and cytoplasmic binding partners. In fact, the PKCα-mediated phosphorylation of Thr 2256 in the NG2 cytoplasmic domain triggers the redistribution of NG2 from apical micro-processes to lamellipodia on the leading edge of the cells, enhancing in turn cell motility (64). Further experimental evidence has revealed that the intracellular C-terminal domain of NG2 plays an important role as an acceptor site for the extracellular signal-regulated kinases (ERK) 1/2 and PKC-α. These interactions activate key signaling pathways involved in cell migration, cell survival, and angiogenesis (64). Moreover, other modifications are possible because NG2 expresses putative proteolytic cleavage sites whose function is still widely unknown (67). However, experimental evidence has suggested that they are involved in neuronal network formation or activated pericyte functions (23, 68). Moreover, NG2 intracellular signaling has been shown to play an important role in the progression of several tumor types where NG2 expression is upregulated, predominantly found in glioblastoma. This correlates with a poor prognosis due to increased NG2-mediated chemo- and radio-resistance of tumor cells (14, 15).

## The NG2 expression

Although there is a remarkable trend toward consistency in a large number of experimental data, there is still no consensus on the NG2 cellular localization. Here, we will not discuss the numerous studies that have examined the expression of NG2 in the CNS. Instead, in the following three sections, we will focus on the few pivotal articles that, according to the authors of this review, represent milestones and have significantly influenced the research in this field. In the 90s, NG2-expressing cells in CNS were typically associated with parenchymal cells, which sparked considerable confusion within the neuroscience community. Studies on parenchymal NG2-expressing cells, usually suggestive of glial cells, generated a lot of misunderstanding among the neuroscience community: on one side, these cells display a combination of features unexpected for glial cells; on the other side, they show features closely related to neurons. These features includes uniform distribution in grey and white matter areas, physical interaction with neuronal cell bodies, and a similar complex stellate morphology (69–72). Moreover, the NG2 expression is associated with cell lineage and with developing or terminally differentiated cell types. From a functional point of view, NG2-expressing cells are described by intense mitotic activity (6), amplified ability to upregulate the NG2 expression (73), and high rate of migratory activity (74). Subsequent studies have supported the presence of NG2 in various post-natal tissues, including bone marrow, smooth muscle, interfollicular epidermis in the skin, musculoskeletal junctions, pancreas, lungs, eyes, heart, and kidneys (21, 26, 27).

### Oligodendrocyte progenitor cells

**Figure 2:**
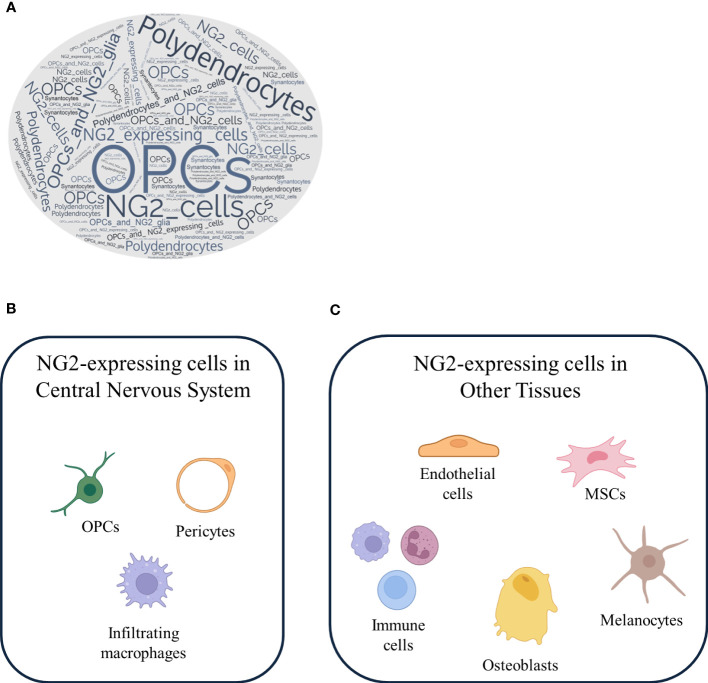
NG2-expressing cells: a versatile cell population with different localization. **(A)** A word cloud generated from PubMed search results for the keywords NG2-expressing cells, OPCs, NG2 glia, oligodendrocytes, and synantocytes reveals the frequency and importance of these terms in the literature. NG2-expressing cells recognized by OPCs are clearly a major focus of research. NG2-expressing cells are a heterogeneous population of cells that are found in both the **(B)** CNS and **(C)** other tissues. In the CNS, NG2-expressing cells are primarily OPCs, which can differentiate into mature oligodendrocytes, pericyte and infiltrating macrophages. In other tissues, NG2-expressing cells include endothelial cells, mesenchymal stem cells, immune cells, melanocytes, and osteoblasts.

Based on our knowledge and in line with the existing literature, in the CNS, NG2 is primarily expressed by a specific group of glial cells known as OPCs (5, 20). In fact, by conducting an in silico analysis, we queried the PubMed database using the terms commonly used in the literature to refer to NG2-expressing cells. As shown in the word cloud in **Figure 2A**, where the size of each word corresponds to its frequency in the queried dataset, the term most frequently employed to describe NG2-expressing cells is “OPCs”. However, as evident, the other terms also enjoy a significant presence in the literature. To support this statement, the paper authored by Kang and colleagues represents the most consistent and reliable evidence confirming that NG2-expressing cells are indeed OPCs. In their manuscript, they investigated the fate of NG2-expressing cells in the normal CNS using genetic lineage tracing *in vivo*. They utilized mice expressing tamoxifen-inducible Cre under the control of the platelet-derived growth factor receptor α (PDGFRα) promoter, an alternative marker for OPCs in a mouse model of amyotrophic lateral sclerosis. They have demonstrated that NG2-expressing cells resident in the CNS differentiate into mature myelinating oligodendrocytes in both the brain and spinal cord during early postnatal and adult life. However, contrary to previous findings (71, 75–78), they have shown that NG2-expressing cells do not give rise to neurons or astrocytes in any region of the brain or spinal cord. Furthermore, clonal analyses have revealed that individual NG2-expressing cells in both regions retain the capacity to proliferate and generate mature oligodendrocytes, suggesting that NG2-expressing cells are not inherently different in their ability to divide and differentiate, as previously discussed. However, Kang et al. (20) did not analyze or report on the fate of NG2-expressing cells at the embryonic level in their study. In fact, other *in vivo* fate mapping studies in mice, have revealed that embryonic, but not adult, NG2-positive cells in the ventral forebrain and hippocampal gray matter differentiate into protoplasmic astrocytes (79–81) (for a detailed discussion, refer to the “NG2-expressing cells: lineage, temporal and spatial restrictions in the CNS” section). Moreover, they have performed fate mapping experiments of NG2-expressing cells in the spinal cord during neurodegeneration. They have revealed that the enhanced proliferation of these progenitors is accompanied by a facilitated differentiation with commitment toward the oligodendrocyte lineage (20). These results imply that NG2-expressing cells are not multipotent progenitors but rather oligodendrocyte progenitor with limited lineage potential, playing a role in the maintenance of the balance of oligodendrocytes in both normal and diseased CNS. Moreover, OPCs typically co-express other OPC markers like the PDGFRα (53, 82), and they are responsible for generating mature and myelinating oligodendrocytes in both the brain and spinal cord (5, 20, 83).

### Pericytes

The other cells known to express NG2 in the CNS are the activated pericytes (9, 19). Stallcup et al. (9), by a mean of immunofluorescence mapping and *in vivo* experiments, have demonstrated that the NG2 proteoglycan is invariably expressed by pericytes, namely the mural cell component of mouse neurovascular units (NVU) in the CNS. Recent technological advances have enabled the identification and visualization of pericytes in live animals by using specific markers. Using transgenic animal models, He et al. have found that cells expressing both PDGFRβ and NG2 markers are identified as vascular mural cells, while cells expressing only one of the two markers are considered other neuroglial cells (79, 84). Pericytes are specialized cells that are found in the walls of capillaries, arterioles, and venules in the brain. They play a crucial role in regulating blood vessel functions and have been implicated in several physiological and pathological processes. One of the primary functions of pericytes is to regulate blood flow through capillaries by contraction or relaxation in response to various stimuli (30). Pericytes also play a role in maintaining the integrity of the NVU, which is essential for protecting the brain from insults. In addition, pericytes have important roles in angiogenesis, the process by which new blood vessels are formed, and they are involved in both the initial sprouting of new blood vessels and their subsequent stabilization and maturation (85). For example, in gliomas and glioblastomas, NG2 is highly expressed in pericytes, and these cells may promote tumor growth and metastasis formation by providing a source of growth factors and promoting neoangiogenesis (86). Additionally, pericytes are recruited to newly formed vessels by glioma cells expressing PDGF-BB (87). Moreover, in diabetic retinopathy, pericytes in the retinal capillaries are primary targets of hyperglycemic damage, which leads to pericyte apoptosis and eventually, vision loss (88). In particular, PDGFRβ expressed by pericytes is dephosphorylated, thus inhibiting the interaction between pericytes and endothelial cells, leading to the development of abnormal blood vessels (89, 90). Recent studies have also shown that pericytes play a role in various neurological diseases, including Alzheimer’s disease (AD), multiple sclerosis (MS), and stroke. In AD, pericytes have been shown to be involved in the clearance of β-amyloid, a key pathological protein in the disease (91). Additionally, the β-amyloid drives pericyte constriction and detachment through the shedding of NG2, thus loosening the connection between pericytes and endothelial cells (92, 93). The loss of pericytes from the capillary wall leads to the loss of blood-brain barrier (BBB) function and cognitive decline. Moreover, pericyte degeneration is accelerated in AD carriers of the apolipoprotein E4 isoform, and this is independent of the β-amyloid pathology (94). Dysfunctional pericytes have also been implicated in the NVU disruption in MS and stroke (95, 96). In MS, the BBB disfunction is associated to pericyte degeneration. In particular, De La Fuente et al. have shown that during normal remyelination, pericytes produce laminin 211 which is involved in OPC maturation; thus the lack or dysfunction of pericytes results in myelin defects (97). Moreover, in the animal model of MS, NG2-expressing pericytes facilitate macrophage infiltration into the brain parenchyma (98). Furthermore, in stoke, pericytes constrict capillaries within 1 hour after ischemia in response to oxidative-nitrative stress (99). This leads to the reduction of blood flow and subsequent pericyte death, resulting in the aggravation of BBB disruption (100).

### Immune cells

Another deeply debated topic stems from the seminal work of Bu et al. in 2001, which emphasized that NG2 is expressed by activated macrophages in kainic acid-induced lesions in the CNS, suggesting a link between NG2 expression and the immune system (33). Several groups have found the expression of NG2 in mouse T cells (101), and our recent finding is consistent with these observations, in which we have revealed that NG2 is expressed by dendritic cells, playing a role in their pro-inflammatory activation (38) (for a detailed discussion, refer to the “The role of NG2 expressing cells in neurological disorders” section). The expression of NG2 on myeloid-derived cells in the CNS, such as macrophages, constitutes another point of challenging scientific interpretation, although Gao et al. (102) have essentially defined that NG2 is expressed to a lesser extent on activated microglia. This seems to be true also for microglia, which are induced to express NG2 following lipopolysaccharide (LPS) injection (102). Moreover, other observations have arisen from a recent finding that NG2 expression can be induced in primary macrophages and in both RAW264.7 and THP-1 cells by the treatment with toll-like receptor ligands, such as poly-I:C or LPS (31). Over the past decade, the concept and terminology of the microglial phenotype polarization have been adapted from the literature on peripheral macrophages; thus, it is not fully accurate to consider these two cell types as overlapping. While microglia share some similarities with macrophages, they exhibit several differences and possess their own unique set of characteristics. Notably, microglia originate from a distinct early yolk sac progenitor population, giving them different developmental origins compared to macrophages. Moreover, once they reside in the CNS, microglia undergo local self-renewal to maintain their population. Under normal conditions, there is no infiltration of peripheral macrophages into the CNS, while during neuroinflammatory diseases, massive macrophage infiltration takes place. Additionally, microglia maintain distinct expression profiles compared to peripheral macrophages, suggesting that microglia are not merely macrophages that have migrated into the brain; instead, they constitute a distinct cell type (103, 104). Furthermore, in response to various pathological conditions, such as neuroinflammation and autoimmune diseases, macrophages from the peripheral immune system can cross the BBB and infiltrate the CNS to engage in the inflammatory response and tissue repair mechanisms (105–107). This infiltration is a component of the immune response that can either produce a protective or a harmful effect. Drawing from these assumptions and in line with our observations in NG2-expressing macrophages (38), it is suggested that microglia do not express NG2, whereas NG2 expression is likely to be present in CNS-infiltrating macrophages. In activated microglia, experimental results have indicated that NG2 mediates the induction of the inducible nitric oxide synthase (iNOS) and the expression of inflammatory cytokine, but not chemokine expression, and it plays a role in their cytotoxicity. In conclusion, the localization of NG2 expression remains a topic of ongoing debate, with evidence pointing to its presence on OPCs, pericytes, and infiltrating macrophages within the CNS (**Figure 2B**) and outside the CNS (**Figure 2C**).

## NG2-expressing cells: lineage, temporal and spatial restrictions in the CNS

NG2-expressing cells are restricted in their lineage, temporal and spatialexpression patterns. In terms of lineage restriction, NG2-expressing cells are restricted to OPCs, meaning that they can differentiate into mature oligodendrocytes but no other glial cell types, such as astrocytes or microglia. In terms of temporal expression, NG2-expressing cells are most abundant during early postnatal development but continue to exist in the adult CNS. In terms of spatial expression, NG2-expressing cells are typically found in areas of the CNS that are undergoing active myelination, such as the corpus callosum and the optic nerve. These cell types, temporal and spatial restrictions suggest that NG2-expressing cells have a highly specialized role in the CNS, specifically in the OPC maturation and consequent myelination processes.

### OPCs during embryonic development

During CNS formation in mammals, OPCs are identified as three distinct progenitor populations temporally and spatially segregated. Throughout embryonic development, different pools of migrating precursors expressing NG2 proteoglycan emerge from the medial and lateral ganglionic eminence and from the telencephalic cortex, before spreading into the whole CNS around birth (108). NG2 expression in these cells appears to be dependent on Sox10 transcription factor and PDGFRα after oligodendrocyte lineage specification (81, 109). In particular, the use of Cre-lox transgenic mice for lineage tracing studies has revealed that NG2-expressing cells emerge at different times and locations from distinct waves of progenitors. The first wave originates from the medial ganglionic eminence (MGE) and the anterior entopeduncular area (AEP) of the ventral forebrain. Around embryonic day 16, these cells become evident in the cerebral cortex and gradually distribute across it, completing the process before embryonic day 18. The second wave emerges from the lateral and/or caudal ganglionic eminences (LGE/CGE), while the third wave originates from the postnatal cerebral cortex. Interestingly, while NG2-expressing cells from the first wave diminish over time, those from the second and third waves survive and spread throughout the brain. Some of these cells eventually differentiate into oligodendrocytes (108). It is worth noting that OPCs originating dorsally exhibit a greater capability to form myelin sheaths compared to their ventrally-derived counterparts. However, the specific differences between these origins lack definitive elucidation (20, 110). Moreover, during spinal cord development, NG2-expressing cells make their first appearance on the ventral side around embryonic day 12.5. These cells originate from Olig2^+^ motor neuron precursor cells (pMN). Interestingly, on the dorsal side, NG2-expressing cells emerge later, approximately on embryonic day 15.5 (108, 111). In addition, the production of OPCs from pMN in the ventral spinal cord is dependent on the activity of the sonic hedgehog signaling pathway, a factor that is notably absent on the dorsal side (112).

### OPCs during adulthood

In adulthood, NG2-expressing cells that arise from the aforementioned sources persist within both the brain and spinal cord. These cells have the intriguing ability to self-renew, ensuring their continued presence (79, 113). Although most of the NG2-expressing cells are directed towards OPCs, testified by the expression of canonical oligodendroglia markers as Sox10, Olig2, and CC1 (114), a consistent portion of NG2-expressing cells retains an immature phenotype in the adult brain, but differs from its embryonic counterpart in migration, cell-cycle length, distribution, and lineage restriction (69). Moreover, NG2-expressing cells populate the white matter (WM) more densely than the grey matter (GM) and show heterogeneous behaviors: in WM, these cells have higher proliferation and differentiation rates than their GM equivalents, which conversely seem to be more quiescent (113). This difference can be attributed to environmental signals as well as to intrinsic mechanisms, such as the desensitization of PDGFRα in GM mediated by high concentrations of the platelet-derived growth factor (PDGF) (115). Moreover, in WM and GM, OPCs show also functional differences regarding the expression of ion channels and neurotransmitter receptors, outlining the existence of a putative NG2-expressing subtype capable of physically contacting neurons and responding somehow to action potentials (74, 116). Despite the absence of apparent macroscopic differences in the morphology and marker expression (such as NG2 and PDGFRα) among NG2-expressing cells across different brain areas, disparities exist in terms of differentiation, proliferation, and expression of voltage-gated and ligand-gated ion channels, among other aspects. NG2-expressing cells in the brain’s WM and GM exhibit distinct characteristics. In the WM of the adult cerebral cortex, a significant portion of NG2-expressing cells undergo differentiation into mature oligodendrocytes. Conversely, in the GM, only a small percentage of cells differentiates, and the majority remains in the NG2-expressing state (83, 117). Different studies have demonstrated variations in the rates of OPC differentiation between different brain areas and age groups. For instance, WM OPCs tend to differentiate at a faster pace than their GM counterparts and younger OPCs differentiate more rapidly than older ones (83, 117, 118). Additionally, transplantation experiments have revealed inherent differences in the differentiation potential between WM and GM (119). When NG2-expressing cells from the WM of adult mice are transplanted into both regions (WM and GM), they exhibit similar differentiation efficiency regardless of the external microenvironment. However, the transplantation of GM NG2-expressing cells into both regions yielded dissimilar outcomes: when transplanted into WM, these cells differentiate into mature oligodendrocytes, but the same transformation is challenging to achieve in the GM. Cell cycle dynamics also contribute to the observed differences; in fact, the cell cycle of NG2-expressing cells not only varies between WM and GM within the same region, but also across different brain regions. Although the percentage of proliferating OPCs is comparable in WM and GM (120), there is a notable distinction: WM OPCs exhibit shorter cell cycles compared to their GM counterpart, and this difference becomes apparent when using markers like BrdU and EdU to identify proliferating cells in postnatal mice. In P21 mice, the cell cycle of WM NG2-expressing cells is approximately 3 days, contrasting with around 19 days in the GM (120, 121). As mice reach P60, the cell cycles for WM and GM NG2-expressing cells is 10 and 36 days, respectively. Furthermore, through analyses of WM in the spinal cord and cerebral cortex, it has been observed that OPCs in the spinal cord’s WM exhibit a cell cycle of around 14.9 days, whereas in the cerebral cortex’s WM, the cycle is approximately 9.5 days. Therefore, GM NG2-expressing cells show an opposing pattern, with a longer cell cycle compared to WM NG2-expressing cells (122). The examination of NG2-expressing cells from the cerebral cortex in a controlled *in vitro* setting has demonstrated significant proliferative differences. These cells were observed to form varying numbers of clone cells, ranging from 40 to 340. This variability suggests intricate differences in their proliferative capacities (123). In terms of regulatory factors, Hill and colleagues have observed that PDGF impacts the proliferation of WM NG2-expressing cells, but its influence is not mirrored in GM NG2-expressing cells (115). The underlying reasons for these variations in proliferative capacity remain uncertain, potentially involving the presence of different subtypes of NG2-expressing cells or microenvironmental influences. Moreover, inherent heterogeneity in NG2-expressing cells across different brain regions leads to distinctions in their sensitivity to external factors within the microenvironment. Several researches have highlighted differences in morphology and gene expression, indicating that GM NG2-expressing cells are less mature compared to their WM counterparts (124). In addition, astrocyte-secreted factors have been observed to decrease the migration ability of WM NG2-expressing cells, while this effect is not as pronounced in GM NG2-expressing cells (124, 125). Furthermore, interferon γ (IFN-γ) has been identified as an inhibitory factor affecting NG2-expressing cells proliferation, differentiation, and branching. Importantly, WM NG2-expressing cells appear to be more responsive to the influence of IFN-γ compared to their GM counterparts (126). These intrinsic differences between WM and GM NG2-expressing cells could offer insight into the disparity in remyelination rates following demyelination. Notably, GM exhibits a higher remyelination rate compared to WM, which could be attributed, at least in part, to these variations in NG2-expressing cell behavior and response (29). Apart from localization and functional characterization, NG2-expressing cells show an adult stem-like phenotype that is of great interest for their differentiation potential.

### Decoding the possible astrogenic or neurogenic potential of NG2-expressing cells

Beyond their well-characterized role in the remyelination process, the scientific community has been debating for years on the possible astrogenic and neurogenic potential of adult NG2-expressing cells. Morphological and nuclear density studies have suggested that NG2-expressing cells may also represent an independent glial population distinct from other cell types in the CNS (22). For what concern astrogenesis, embryonic NG2-expressing cells isolated from the optic nerve generate astrocytes or oligodendrocytes depending on the *in vitro* culture conditions (127). More recently, *in vivo* fate mapping studies on BAC-transgenic NG2-CreERT^2^ mouse line have shown protoplasmic astrocyte differentiation of embryonic but not adult NG2-expressing cells in ventral forebrain and hippocampal GM (128), also confirmed by the presence of NG2-derived astrocytes in the ventral cortex and other brain regions (114, 129). From these studies, the astrogenic potential of NG2-expressing cells appears to be limited to the embryonic and prenatal stages of CNS development and is lost at the postnatal stage (114). Interestingly, the reduction of cell lineage plasticity and oligodendrocyte commitment of NG2-expressing cells are reported to be gradually achieved around birth (130), although these cells possibly retain some degree of astrocytic differentiation potential visible under certain conditions, as during CNS insults (131). However, the typical marker of astrocytes glial fibrillary acidic protein (GFAP) is expressed in only 20% of all NG2-expressing cells, and it is only seen within a limited time window (132). As for astrocyte phenotype, lineage tracing techniques have been also used to study the neurogenic potential of NG2-expressing cells. Indeed, one of the most interesting theories about neuron-NG2-expressing cell communication relies on the integration of synaptic inputs by NG2-expressing cells and subsequent modulation of different activation programs. For instance, network activity changes can promote oligodendrocyte maturation for myelin remodeling and replacement (133), as well as response after tissue damage (132, 134). Moreover, studies using Plp-Cre-ERT2/Rosa26-EYFP (PCE/R) double-transgenic mice have depicted early postnatal NG2 progenitors as multipotent cells able to produce HuC/D^+^ immature glutamatergic neurons in dorsal and ventral forebrain (135). Similar results have been also obtained on adult NG2-expressing cells, reported to produce cortical Pax6^+^/Sox2^+^ glutamatergic progenitors in the piriform cortex and HuC/D^+^/NeuN^+^ neurons in the hypothalamus with immature electrical properties (113, 136). By contrast, lineage tracing studies on Olig2-CreER™ and PDGFRα-CreER transgenic mice and immunohistochemical analysis of NG2 expression in the postnatal subventricular zone have shown no evidence of neurogenesis (5, 137). In conclusion, while NG2-expressing cells exhibit some limited astrogenic potential during embryonic development, this capability is largely absent in adulthood, and the neurogenic potential of NG2-expressing cells in the adult CNS appears to be unsupported by current evidences.

## The role of NG2-expressing cells in neurological disorders

### Neuroinflammation

The hypothesis that NG2-expressing cells could play a role during inflammation has arisen from the observation that, in the CNS, they express receptors for inflammatory cytokines (6, 138, 139). Here we will explore the more recent data about the role of NG2 in the inflammatory response in the CNS, mainly focusing on MS and its animal model, the experimental autoimmune encephalomyelitis (EAE). Similarly to the lineage potential definition (for a detailed discussion, refer to the “NG2-espressing cells: lineage, temporal and spatial restrictions in the CNS” section), following CNS insults, the role of NG2-expressing cells appears to be controversial and highly influenced by different cell types and by the surrounding inflammatory environment. As previously demonstrated, NG2-expressing cells in the adult rat cerebellum are the major mitotic cell type and represent a significant part of the total number of cells in the CNS (140, 141). NG2 expression has been reported in many principal actors of the inflammatory response and only during neuroinflammation, such as in OPCs, pericytes, monocytes, macrophages, dendritic cells, and microglia. However, some authors have suggested that NG2 positivity in some cell types may be only transient or the result of cellular activities, such as phagocytosis, and thus may not be correlated with proper NG2 protein expression (38, 142–146) (for a detailed discussion, refer to the “NG2 expression” section). The importance of NG2 is highlighted by the fact that, following brain damage, there is an overall increase in its expression level accompanied by the rapid activation of cell types known to express it (76, 147, 148). For instance, NG2 expression in the immediate surroundings of a puncture lesion in the cerebellum has been seen to increase over a period of 7 days, in parallel with microglia activation and significant NG2-expressing cell proliferation and migration into the brain parenchyma (144, 149). Moreover, it has been reported that NG2-expressing cell hyper-reactivity occurs after a wound or kainite-induced lesions. This results in an overall increase of NG2 expression and also in a positive modulation of NG2 production in single cells. Similarly, these findings suggest that NG2-expressing cell hyper-reactivity occurs in response to certain types of injury (143, 150). The accumulation of NG2 in the lesion site can have several possible explanations. Among others, OPCs are the best-characterized NG2-expressing cell population in the adult brain (for a detailed discussion, refer to the “NG2 expression” section) for their ability to mature into adult oligodendrocytes for adaptive myelination or remyelination. After brain injuries, OPCs can activate proliferation, migration, and differentiation programs for proper tissue reaction (151). In line with these observations, NG2 has been observed to stimulate OPC proliferation and promote oligodendrocyte differentiation, albeit without a discernible correlation between the two processes (152, 153). This may lead to an increased number of OPCs which are not necessarily translated into a proportional number of mature NG2^-^ oligodendrocytes for proper injury repair. Moreover, OPC differentiation inhibitory potential has been attributed to several inflammatory cytokines commonly found in lesioned environments, such as TNFα (154), IL-17 (155), and INFγ (126, 156). This strengthens the idea of an unbalanced ratio between NG2-expressing OPCs and NG2^-^ oligodendrocyte concentration during inflammation. Quantification of NG2 by immunofluorescence techniques has revealed that the accumulation of OPCs in the lesioned area can contribute to the formation of glial scars, which have been reported to hinder both structural and functional axon regeneration (157). Interestingly, after NG2 shedding mediated by enzymes such as metalloproteases MMP-13 and MMP-14, NG2 labeling within the glial scar can also follow the incorporation of its extracellular portion in the extracellular matrix (158, 159). According to Wennstrom and co-workers, the NG2 shedding process results in the production of a soluble portion of NG2 (for a detailed discussion, refer to the “Structure and functional domains of NG2” section) (160, 161). This form of NG2 appears to increase in concentration following systemic inflammation, and it has been reported to possess inhibitory action on neuronal outgrowth similar to its protein of origin (162–164). Another possible explanation for increased NG2 expression during inflammation can be related to a facilitated infiltration of NG2^+^ immune cells, like macrophages, dendritic cells, and T cells, from the blood stream into the injured CNS due to the increased BBB permeability. It is well established that pericytes are important modulators of BBB integrity, and that during inflammation endothelial cell tight junctions of the BBB can progressively be disrupted, causing BBB leakage and immune cell infiltration in the brain parenchyma (for a detailed discussion, refer to the “NG2 expression” section). We have previously reported that NG2 knock-out (NG2KO) mice show more regular and preserved claudin-5/occludin patterns (structural proteins of BBB) with respect to wild-type (WT) cells (38). This is accompanied by a reduction in the perivascular extravasation of leukocytes in NG2KO mice and an improvement in several parameters of EAE, including disease incidence, onset time, and severity score (38). Along this line, selective NG2 ablation on myeloid cells was reported to strongly reduce the recruitment of CD18^+^ cells in the lesion site with less pronounced myelin damage (6). Similar results have also been obtained by selective ablation of NG2 expression in tumor macrophages, resulting in impaired recruitment of CD18^+^ cells to the tumor region, and producing an extensive delay in tumor progression (165). More recently, NG2KO OPCs have been shown to be less associated with blood vessels and NVU during EAE than WT OPCs. Therefore, WT OPCs have been reported to be closely related to BBB regions, particularly dysfunctional tight junctions or altered claudin-5/occludin patterns (19). The absence of NG2 reduces the responsiveness to extracellular recruitment factors, like vascular endothelial growth factor A (VEGF-A) or transforming growth factor β (TGF-β), and it may interfere with intracellular mediators of cellular motility, like syntenin-1. This reduced association with the BBB can be attributed to the reduced migration of NG2KO OPCs (19, 56). Besides the NG2 role on OPCs and immune cells, NG2-expressing cells also crosstalk with resident microglia, the major cell type contributing to inflammatory response in the CNS. The relationship between NG2-expressing cells and microglia is mediated by multiple factors, such as TGF-β family members, which are implicated in shaping microglia activation and NG2 expression under inflammatory conditions. Specifically, an increase in NG2 expression has been observed following intracerebral injection of TGF-β, resulting in an enhanced activation of microglia. This activation of microglia further facilitates the NG2 signal through the secretion of TGF-β1 (166, 167). NG2-expressing cells can themselves modulate microglia activation by the secretion of TGF-β2, acting on microglial TGF-β2 receptor-CX3CR1 signaling pathway (168). TGF-β2 has been extensively characterized as a potent inhibitor of microglia activation and proliferation (169), and this mechanism has been associated with NG2 intracellular signal in NG2-expressing cells (170). Ablation of NG2 causes massive pro-inflammatory activation of microglia, accompanied by upregulation of pro-inflammatory and phagocytic mediators and downregulation of checkpoint genes responsible for the maintenance of microglia homeostasis. In contrast, the potent downregulation of the CX3CR1 gene is induced by selectively silencing NG2 in activated microglia. This downregulation is almost completely reversed by the administration of TGF-β2 or by the use of a TGF-β2 receptor agonist, emphasizing the significance of the TGF-β2-TGFR-β2-CX3CR1 regulatory axis (171). Apart from the TGF-β family, NG2-mediated control of microglia has been reported for other signaling regulators, such as the hepatocyte growth factor (HGF), already characterized as a specific product of OPCs and neurons (172). NG2 ablation in proliferating OPCs in NG2-HSVtk transgenic mouse model has produced massive apoptotic neuronal cell death in the hippocampus following microglia activation, upregulation of proinflammatory IL-1β, IL-6, and TNFα cytokines, and induction of caspase-dependent apoptosis (18). Moreover, even a subpopulation of microglia has shown NG2 expression and responsiveness to CNS injuries involving the regulation of NG2. NG2 expression was not reported in postnatal and adult OX-42^+^ microglia cells except after LPS administration when NG2 positivity is accompanied by increased iNOS expression and Il-1β production. This effect can be reversed by silencing NG2 by siRNA transfection (102, 173). Furthermore, the increase in neurotrophic factors, such as nerve growth factor and glial-derived growth factor, has also been documented and linked with the decrease in NG2-dependent phosphorylation of FAK. The activity of FAK in an inflammatory setting seems to establish a feedback loop, amplifying and propagating inflammation along with the cytokine TNFα (173, 174). Taken together, these data clearly show that NG2 \and NG2-expressing cells plays an important role in neuroinflammation. Moreover, NG2 appears as a bivalent regulator of brain homeostasis, being a neuronal survival promoter under normal conditions, or an inflammatory enhancer and neuronal regeneration inhibitor in an inflammatory environment. Such ambiguous activity needs to be further characterized for a better understanding of NG2-specific pathways and for evaluating potential therapeutic applications in neuroinflammation involving NG2 modulation. In this section, we have summarized studies on NG2-expressing cells and their involvement in neuroinflammation, highlighting their multifaceted and context-dependent roles. Under certain conditions, these cells can act as promoters of inflammation, yet they may also support neuronal survival to a lesser extent in other contexts. However, we suggest that the NG2-expressing cells tend to promote neuroinflammation and inhibit neuronal regeneration in inflammatory CNS.

### Neurodegeneration

NG2-expressing cells have emerged as significant players in the pathophysiology of neurodegenerative diseases such as Parkinson’s disease (PD), Alzheimer’s disease (AD), and Amyotrophic Lateral Sclerosis (ALS). NG2-expressing cell involvement in neurodegeneration is multifaceted and varies significantly across different conditions, being related to neuron-glia interactions, myelin maintenance, and response to neural injury. For PD, Zhang SZ et al. have shown that in the neurotoxin 1-methyl-4-phenyl-1,2,3,6-tetrahydropyridine (MPTP)-induced mouse model, NG2-expressing cells have a neuroprotective function on dopaminergic neurons via TGF-β2 signaling. Moreover, these cells play a crucial role in maintaining the resting state of microglia and suppressing their over-activation induced by the MPTP neurotoxicity. The authors have found that the deficiency of NG2-expressing cells contributes to both neuroinflammation and nigral dopaminergic neuron loss (175). In addition, in a rat model of PD, it has been found a decrease in the number of NG2-expressing cells in the substantia nigra (176). Other studies have shown an association of OPCs and oligodendrocytes to PD (177), particularly with PD-linked risk loci (LRRK2 and GBA1) involved in the regulation of different molecular pathways leading to the development of the disease (178). Studies on another PD-like neurodegenerative disorder, the multiple system atrophy (MSA), have shown that there are abnormal cytoplasmic accumulations of α-synuclein only in oligodendrocytes but not in OPCs (179). These results were obtained in both *in vitro* and *in vivo* settings, in an experimental rat model of inflammatory demyelination showing an increased expression of α-synuclein (180). Moreover, in a mouse model of MSA, this accumulation in oligodendrocytes causes myelin degeneration, axonal loss and gliosis (181). In AD and ALS, experimental data have shown that impairments in oligodendrocyte metabolism and myelin integrity frequently precede the clinical onset of neurodegeneration (182, 183). In AD, the amyloid-β peptides accumulate and are deposited around neurons, forming amyloid plaques. In the 3xTg-AD mouse model, OPCs have been shown to display morphological changes, including atrophy and hypertrophy. The OPC disruption is an early pathological sign in AD, and this is a potential factor in accelerating myelin loss and cognitive decline (184). In addition, also in the APP/PS1 mouse model, myelin abnormalities appear before the onset of overt disease symptoms, suggesting an effect of amyloid plaque deposition or mutated neurons on OPCs (182). Moreover, post-mortem studies have shown that OPCs are disrupted in AD, leading to myelin loss and failure of regeneration, with consequent white matter damage (185). In particular, this occurs in about 50% of all AD cases and seems to be due to glutamate excitotoxicity, oxidative stress, and direct toxic effect of amyloid-β peptides (186, 187). In addition, in the CSF of AD patients, levels of soluble NG2 decrease, in line with a loss of cells of the oligodendrocyte lineage. The authors have suggested that this could be due to an effect of the amyloid-β aggregation, possibly leading to inhibition of NG2 shedding or downregulation of its expression (188). Studies on the progressive supranuclear palsy (PSP), a neurodegenerative disease in which there is the accumulation of microtubule-associated tau protein, showed an increase of tau protein only in oligodendrocytes but not in OPCs. These oligodendrocytic accumulations of tau protein form coiled bodies and lead to myelin loss (179). Furthermore, in ALS, there is an abnormal accumulation of insoluble and misfolded proteins in motor neurons, leading to their degeneration and death (20). Studies carried out on the G93A mouse models with superoxide dismutase 1 (SOD1) mutations have shown that there is an enhanced proliferation and differentiation of OPCs, resulting in extensive gliosis. Despite the ongoing degeneration and death of oligodendrocytes, the overall number of oligodendrocytes remains unchanged, highlighting the critical role of OPCs in trying to mitigate the loss of oligodendrocytes, although their compensatory mechanisms are limited under pathological conditions (20, 189). However, the new oligodendrocytes fail to mature adequately, which negatively impacts both their myelination capacity and their ability to provide metabolic and trophic support to axons, contributing to further motor neuron death (190, 191). Moreover, alterations in myelin structure are detectable in presymptomatic spinal cords, while in symptomatic stages, the morphological and biochemical myelin degeneration increases (192). Furthermore, studies in ALS animal models have reported the disruption of the lactate transport and the increase of iron levels in the extracellular environment due to oligodendrocyte damage, as contributors in neuronal loss (193, 194). Overall, NG2-expressing cells are integral to the pathophysiology of neurodegenerative diseases. It appears that the NG2-expressing cells response is more closely tied to demyelination and to the specific context of the degenerative process, rather than to neuronal loss itself. In addition, NG2-expressing cells are central to the neural response to injury and disease, potentially offering novel therapeutic targets for mitigating neurodegenerative damage and promoting CNS recovery (183).

## Conclusions

NG2-expressing cells have different functions in the adult CNS and other tissues. They participate in complex signaling networks through interactions with various extracellular and intracellular binding partners. Understanding the precise role of NG2 in different physiological and pathological contexts will contribute to elucidate its potential as a therapeutic target in neuroinflammatory and neurodegenerative diseases. While our review has outlined the multifaceted roles of NG2-expressing cells in the CNS, there are several avenues for further exploration and development. Future research could benefit from proposing specific hypotheses and experimental approaches to address the remaining questions and to propel the field forward. For instance, investigating the regulatory mechanisms that govern NG2-mediated neuroinflammation and its implications for CNS disorders could provide valuable insights. Furthermore, given that OPCs seem to exhibit regional functional differences, it is plausible to speculate that distinct populations of NG2-expressing OPCs exist within the CNS parenchyma. To explore this possibility, spatial transcriptomic experiments could be highly informative, revealing potential subpopulations of OPCs based on their unique mRNA expression patterns. This could also lead to definitive evidence that NG2 expression is restricted to OPCs in the CNS parenchyma. NG2-expressing cells in CNS parenchyma have been shown to have the potential to differentiate into oligodendrocytes, the cells responsible for myelinating axons in the CNS. Convincing *in vitro* and *in vivo* experiments have demonstrated that NG2-expressing cells can differentiate into oligodendrocytes in response to growth factors, such as PDGF and bFGF. Further research is needed to better understand the specific pathways and mechanisms involved in NG2-mediated regulation of neuroinflammation. Such understanding may pave the way for potential therapeutic applications targeting NG2 modulation in neuroinflammatory disorders. Moreover, expanding the studies on the therapeutic potential of targeting NG2-expressing cells in various CNS disorders is crucial. Understanding how NG2-expressing cells contribute to disease pathogenesis and how they can be therapeutically modulated holds promise for novel treatment approaches. There are currently no anti-NG2 therapies approved for the use in humans, but there is a clinical trial (NCT06096038) underway to test the safety and efficacy of T cells expressing CSPG4-specific Chimeric Antigen Receptors (CAR) in persons with head and neck squamous cell carcinoma. Indeed, a more thorough exploration of potential therapeutic strategies targeting NG2 modulation, along with their limitations and possible enhancements, could provide significant benefits for persons with neurological disorders. The NG2-expressing cells exhibit spatial and temporal restrictions, lineage specificity, and functional differences in different regions of the CNS. Moreover, they appear to have additional roles: under normal conditions, they promote neuronal survival and homeostasis, and in an inflammatory environment, they can increase inflammation, inhibit neuronal regeneration, and contribute to the formation of glial scars. Accordingly, while their differentiation potential beyond oligodendrocytes is quite accepted, they likely play important roles in modulating neuronal activity and responding to CNS insults. A mix of intrinsic and extrinsic variables, including epigenetic changes, signaling molecules, and environmental cues, controls the biological potential of NG2-expressing cells. Further research is needed to fully understand the complex functions and potential of NG2-expressing cells in the CNS.

## Author contributions

MB: Writing – review & editing, Data curation. GP: Data curation, Writing – review & editing. CB: Data curation, Formal analysis, Writing – review & editing, Writing – original draft. TV: Formal analysis, Writing – review & editing. AU: Supervision, Writing – review & editing. GF: Conceptualization, Writing – original draft, Writing – review & editing.
